# Using diffusion tensor imaging to depict myocardial changes after matured pluripotent stem cell-derived cardiomyocyte transplantation

**DOI:** 10.1016/j.jocmr.2024.101045

**Published:** 2024-05-23

**Authors:** Moses P. Cook, Wahiba Dhahri, Michael A. Laflamme, Nilesh R. Ghugre, Graham A. Wright

**Affiliations:** a Department of Medical Biophysics, University of Toronto, ON, Canada; b McEwen Stem Cell Institute, University Health Network, Toronto, ON, Canada; c Peter Munk Cardiac Centre, University Health Network, Toronto, ON, Canada; d Department of Laboratory Medicine and Pathobiology, University of Toronto, ON, Canada; e Schulich Heart Research Program, Sunnybrook Research Institute, Toronto, ON, Canada; f Physical Sciences Platform, Sunnybrook Research Institute, Toronto, ON, Canada

**Keywords:** Magnetic resonance imaging, Diffusion tensor imaging, Pluripotent stem cells, Cell transplantation

## Abstract

**Background:**

Novel treatment strategies are needed to improve the structure and function of the myocardium post-infarction. In vitro-matured pluripotent stem cell-derived cardiomyocytes (PSC-CMs) have been shown to be a promising regenerative strategy. We hypothesized that mature PSC-CMs will have anisotropic structure and improved cell alignment when compared to immature PSC-CMs using cardiovascular magnetic resonance (CMR) in a guinea pig model of cardiac injury.

**Methods:**

Guinea pigs (n = 16) were cryoinjured on day −10, followed by transplantation of either 10^8^ polydimethylsiloxane (PDMS)-matured PSC-CMs (n = 6) or 10^8^ immature tissue culture plastic (TCP)-generated PSC-CMs (n = 6) on day 0. Vehicle (sham-treated) subjects were injected with a pro-survival cocktail devoid of cells (n = 4), while healthy controls (n = 4) did not undergo cryoinjury or treatment. Animals were sacrificed on either day +14 or day +28 post-transplantation. Animals were imaged ex vivo on a 7T Bruker MRI. A 3D diffusion tensor imaging (DTI) sequence was used to quantify structure via fractional anisotropy (FA), mean diffusivity (MD), and myocyte alignment measured by the standard deviation of the transverse angle (TA).

**Results:**

MD and FA of mature PDMS grafts demonstrated anisotropy was not significantly different than the healthy control hearts (MD = 1.1 ± 0.12 × 10^−3^ mm^2^/s vs 0.93 ± 0.01 × 10^−3^ mm^2^/s, p = 0.4 and FA = 0.22 ± 0.05 vs 0.26 ± 0.001, p = 0.5). Immature TCP grafts exhibited significantly higher MD than the healthy control (1.3 ± 0.08 × 10^−3^ mm^2^/s, p < 0.05) and significantly lower FA than the control (0.12 ± 0.02, p < 0.05) but were not different from mature PDMS grafts in this small cohort. TA of healthy controls showed low variability and was not significantly different than mature PDMS grafts (p = 0.4) while immature TCP grafts were significantly different (p < 0.001). DTI parameters of mature graft tissue trended toward that of the healthy myocardium, indicating the grafted cardiomyocytes may have a similar phenotype to healthy tissue. Contrast-enhanced magnetic resonance images corresponded well to histological staining, demonstrating a non-invasive method of localizing the repopulated cardiomyocytes within the scar.

**Conclusions:**

The DTI measures within graft tissue were indicative of anisotropic structure and showed greater myocyte organization compared to the scarred territory. These findings show that MRI is a valuable tool to assess the structural impacts of regenerative therapies.

## 1 Introduction

Myocardial infarction (MI) remains the most common cause of heart failure (HF) worldwide [1]. HF is a debilitating disease that has a mortality rate of ∼50% over 5 years [2]. This challenge is further compounded by a lack of curative strategies, so clinicians resort to treating patients’ symptoms. While medical therapy can slow the progression of the disease, it cannot replace the cardiomyocytes lost irreversibly as a result of the ischemic insult to restore heart function. Heart transplantation remains the gold standard for treatment, offering hope for only 10% of highly selected candidates [3]. Novel myocardial cell-based therapies, such as implantation of mesenchymal stem cells [4], bone marrow-derived cells, [5] and pluripotent stem cell-derived cardiomyocytes (PSC-CM) [6], [7], offer a promising means of restoring contractile function to the infarcted heart. Recently, Dhahri et al. [8] have demonstrated an economical method to mature PSC-CMs in vitro using polydimethylsiloxane (PDMS). Their study was the first to showcase the successful engraftment of adult-like cardiomyocytes to the scarred region in a guinea pig model of cardiomyocyte injury. Furthermore, the matured PDMS PSC-CM graft had improved electromechanical integration, sarcomeric organization, and enhanced contractility compared to its immature tissue culture plastic (TCP)-matured graft counterpart. It follows that structural characteristics of healthy myocardium [9] must be mirrored in the graft to create force-generating units, a right-handed (RH) to left-handed (LH) helical myocyte orientation, and aligned myocytes. Importantly, imaging biomarkers can help elucidate these structures.

One method for the evaluation of structural and functional benefits post-therapy is cardiovascular magnetic resonance (CMR), as it allows for the in vivo quantification of cardiac function [10] and viability [11]. Previous studies have employed in vivo CMR to quantify outcomes post-non-myocyte therapy, specifically using diffusion tensor imaging (DTI) to quantify host remodeling and anisotropy of healed myocardium [12], [13]. In the context of PSC-CM therapy, DTI would be an invaluable tool to probe the host and graft structural characteristics mentioned previously. Using a subset of ex vivo subjects from the companion study [8], we tested the hypothesis that mature PDMS grafts will have improved cell alignment and anisotropic structure in DTI when compared to immature TCP grafts in a guinea pig model of myocardial injury.

## 2 Methods

### 2.1 PSC-CM preparation and maturation

PSC-CMs were derived from the protocol as previously described [8]. In sum, pluripotent ESI-17 human embryonic stem cells (Biotime, Carlsbad, California) were differentiated into the cardiomyocyte lineage. First, cell aggregates were treated with bone morphogenetic protein-4, Wnt inhibitor IWP2, and activin A in suspension culture. Single cells were then plated onto either TCP or roller bottles lined with 112 µm films of PDMS to derive them into immature or mature PSC-CMs, respectively.

### 2.2 Animal preparation

**Fig. 1:**
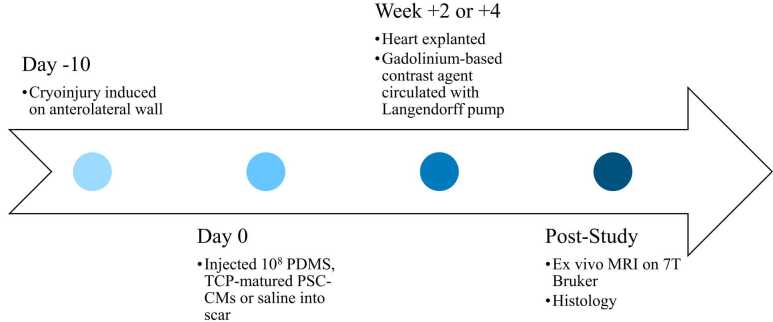
Timeline of subject preparation. The experimental protocol begins with scar initiation via cryoablation, followed by injection of either polydimethylsiloxane (PDMS), tissue culture plastic (TCP)-matured pluripotent stem cell-derived cardiomyocytes (PSC-CMs) or pro-survival cocktail alone (n = 6, n = 6, n = 4, respectively) 10 days later. At either week +2 or week +4 post-transplantation, hearts were explanted and prepared for subsequent MRI. Histological validation was then performed post-MRI. MRI: magnetic resonance imaging.

Twenty male Hartley guinea pigs (Charles River Laboratories, Wilmington, Massachusetts) were acquired post-mortem from the companion study, where the detailed methodology regarding PSC-CM maturation and transplantation is described [8]. In short, animals were cryoinjured on the anterolateral wall via thoracotomy on day −10 (Fig. 1). Cryoinjury was induced with the use of an 8-mm diameter aluminum probe which was then cooled using liquid nitrogen. It was then applied to the epicardium four times each for 30 seconds. All animals underwent immunosuppression starting day −2. Next, transplantation was performed transepicardially by a second thoracotomy of either 10^8^ PDMS-matured PSC-CMs (n = 6) or 10^8^ TCP-matured PSC-CMs (n = 6) on day 0. Vehicle (sham-treated) controls were injected with a pro-survival cocktail made of growth factor-reduced Matrigel (Corning, Corning, New York), 50 µM of Pinacidil (Sigma, St. Louis, Missouri), 100 ng/mL insulin-like growth factor-1 (PeproTech, Cranbury, New Jersey), and 200 nM of Cyclosporine A (Novartis/Sandimmune, Basel, Switzerland) without PSC-CMs (n = 4), and healthy controls did not undergo treatment (n = 4). Immediately before sacrifice at either week +2 (n = 3 TCP-matured, n = 2 PDMS-matured) or week +4 (n = 3 TCP-matured, n = 4 PDMS-matured), animals were injected with 0.3 mL/kg body weight of the gadolinium contrast agent gadobutrol (Bayer Healthcare, Mississauga, Ontario, Canada) intraperitoneally 30–60 minutes before sacrifice. Subjects underwent ex vivo optical mapping at end-study and were arrested using blebbistatin (15 μM, Cayman Chemical, Ann Arbor, Michigan). Since contraction is inhibited by blebbistatin [14], all muscles would have relaxed by ex vivo CMR. Finally, hearts were perfusion fixed [8] and infused with a second dose of the gadolinium contrast agent to allow for accumulation in the injured region for ex vivo imaging. Gadolinium was injected twice to provide sufficient circulation of the contrast agent throughout the animal. Finally, hearts were then transported to another institution for subsequent ex vivo MRI.

### 2.3 Contrast-enhanced magnetic resonance imaging of ex vivo hearts

Hearts from all animals were scanned on a 7T Bruker Ascend 300WB MRI (Bruker, BioSpin, Billerica, Massachusetts) equipped with a 1500 mT/m gradient using a cylindrical Micro2.5 probe in quadrature. Imaging was run on ParaVision 6.0.1. Hearts were fixed in a 4% buffered paraformaldehyde solution for 3–12 hours, immersed in saline, and held in a vertical position using a custom three-dimensional (3D) printed holder inside a 50 mL Falcon tube.

To characterize scar, contrast-enhanced magnetic resonance (CE-MR) acquisitions were performed using a two-dimensional T1-prepared fast low angle shotproduct sequence from Bruker with inversion recovery using the following parameters: repetition time/echo time (TR/TE), 710/2.8 ms; spatial resolution, 0.192 × 0.192 × 0.5 mm^3^; inversion time, 150–500 ms adjusted to null myocardium. Scar was defined where the signal intensity was >5 standard deviations from the remote tissue [15]. The remote territory was defined as segments 9 and 10 of the American Heart Association 17-segment model [16]. High-resolution CE-MR images were used to compare scar and graft regions to the ground-truth histology images. CE-MR image volumes were downsampled and registered to the corresponding diffusion-weighted volumes using an affine transformation in MATLAB (MathWorks, Natick, Massachusetts) to derive regions of interest (ROIs) for scar DTI characterization.

Graft tissue ROIs were first identified in the ground-truth histological images based on immunostaining of the human-specific nuclear marker Ku80 [6], [17]. Images were aligned to MRI using the papillary muscles as anatomical landmarks. Graft tissue was then labeled in the CE-MR images using a semi-automatic region-growing algorithm [18] in areas of signal attenuation where graft was expected since graft tissue did not take up the exogenous contrast agent. These ROIs were then used for DTI parametric quantification. ROIs of PDMS and TCP grafts had no significant differences in volume (p = 0.3), and thus both groups were assumed to be equally affected by boundary effects.

### 2.4 DTI of ex vivo hearts

A 3D spin echo, multi-shot echo planar imaging diffusion product sequence from Bruker was used with the following parameters: TR/TE, 1000/19.7 ms; spatial resolution, 0.3 mm^3^ isotropic; scan time, 4 hours; averages, 1; field-of-view, 30 mm × 30 mm × 15.3 mm; matrix size, 100 × 100 × 51; bandwidth, 200 KHz; b-values, 0 and 700 s/mm^2^; 5 b0 images; and 30 diffusion directions. Temperature was maintained at 21°C by thermal conduction of a water tube inside the magnet bore close to the sample. DTI analysis was performed using the Python Dipy Library [19] on 10 slices of the mid-left ventricle (LV).

### 2.5 Image analysis

#### 2.5.1 Anisotropy metrics

Intragroup DTI parameters, including fractional anisotropy (FA) and mean diffusivity (MD), of scar, graft, and remote regions were compared in the treated groups to elucidate changes in myocyte organization post-therapy according to the above definitions. Intergroup differences in graft structure were then compared between TCP, PDMS, and healthy control tissue to test the hypothesis that PDMS grafts will exhibit a more natural myocyte anisotropy. Finally, scar, graft, and remote DTI parameters were each pooled to characterize the anisotropy of graft and healthy tissue, irrespective of cell maturation protocol. Areas of the control hearts were matched to the comparable regions of the treated group. For instance, the anterior portion of the control cohort was analyzed when comparing diffusion parameters to the grafted area of the treated cohort.

#### 2.5.2 Structural metrics

Helix angle (HA), transverse angle (TA), and absolute secondary eigenvector angle (E2A) were defined as per established definitions [20], [21]. Helix angle transmurality (HAT) was calculated by sampling HA values of graft ROIs across the transmural depth (TD) using a linear least squares fit. A histogram of the graft HA values across the TD of the myocardium was binned into 15% intervals from the endo- to epicardial borders. Grafts were generally localized to the mid-myocardium, so data from the first 15% and the last 25% of the TD were omitted. RH, circumferential, and LH cardiomyocyte orientations were defined as voxels with HA values from +30° to +90°, −30° to +30°, and −90° to −30°, respectively. The standard deviation of the TA within graft ROIs was evaluated as a marker of myocyte alignment. Tractography of the graft and whole heart was performed in DSI Studio (https://dsi-studio.labsolver.org) using primary eigenvectors imported from the reconstructed diffusion tensors. Graft tracts were limited to the maximum length of the ROI so as to not include myocytes from the adjacent region.

### 2.6 Histological analysis

Preparation of histological sectioning and subsequent staining was completed as previously reported [8]. In sum, hearts were excised at end-study, fixed via a 4% buffered paraformaldehyde solution, and embedded in paraffin. Hearts were sliced along the short axis every 2 mm resulting in 5–6 sections of 4 µm. Finally, immunostaining for the graft region was performed using the human-specific Ku80 antigen [6], [17] (Cell Signaling, Danvers, Massachusetts). Scar was demarcated by staining with fluorescein isothiocyanate-conjugated collagen hybridizing peptide (F-CHP, 3Helix, Salt Lake City, Utah ) while host ventricular muscle was identified by β myosin heavy chain. Microscopy images were captured using a Zeiss LSM700 laser scanning confocal microscope with either a Plan-Apochromat 63×/1.4 NA or FLUAR 10×/0.50 NA, Plan-Apochromat 20×/0.8 NA, Plan-Apochromat 40×/1.4 NA objective.

Histological myocyte post-processing of the microscopy images was done using the Fiji distribution of ImageJ [22]. Quantification of directionality was performed as highlighted in the companion paper’s supplemental methods [8]. In brief, at least three randomly selected regions in each graft were chosen and averaged using the directionality plugin. DTI-derived primary myocyte eigenvector maps were compared to the corresponding histological ground-truth myocyte orientation by rotating short-axis DTI vector maps to align with the corresponding slice of the histological image containing the graft using anatomical landmarks, such as the papillary muscles, size/shape of the left and right ventricles, and percent distance from the apex as histological slicing, and DTI were performed from apex to base. Primary and secondary myocyte vector angle averages were then calculated within the voxels containing the corresponding histological ROIs. Histology and DTI metrics for the remote [23] and scar [24] territories were not analyzed, as past studies have shown good correlation.

### 2.7 Statistical analysis

Unless otherwise stated, all analyses pooled the +2 and +4 week subjects due to a limited number of subjects in each cell-treated cohort at each timepoint. All bar plots are reported as mean ± standard error of the mean. Intragroup analyses were conducted using a one-way analysis of variance (ANOVA) to identify differences in MD and FA within PDMS graft, TCP graft, and healthy control samples, while a paired t-test was used to determine the significance between the scarred and remote regions of the vehicle cohort. Intergroup analyses were performed using a one-way ANOVA with the Tukey honest significant difference post-hoc test when comparing multiple groups (PDMS vs TCP grafts vs control). A p-value <0.05 was considered statistically significant.

Box plots depict the median of the data, where the box extends to the inner quartiles of the dataset, and whiskers depict the minimum and maximum of the data, excluding outliers. Histological and DTI myocyte directionality differences were shown via Bland-Altman analysis, while correlation and agreement were demonstrated via the coefficient of determination (*R*^2^) and Pearson's correlation coefficient (*r*) using the NumPy package v1.21.5 in Python v3.7.3.

## 3 Results

### 3.1 PDMS-matured PSC-CM grafts demonstrate anisotropic structure

**Fig. 2:**
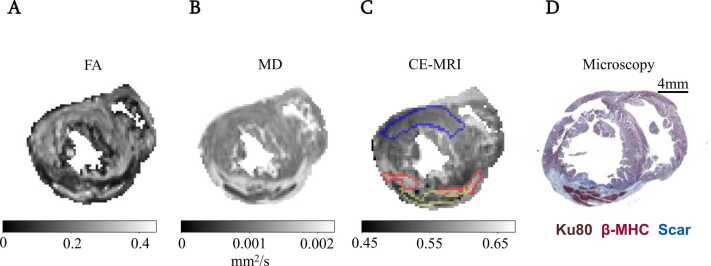
PSC-CM grafts form anisotropic tissue as shown by DTI. (A) Fractional anisotropy (FA) map of polydimethylsiloxane (PDMS)-matured pluripotent stem cell-derived cardiomyocyte (PSC-CM) treated heart in short-axis view. (B) Mean diffusivity (MD) map. (C) Co-registered contrast-enhanced (CE) T1-weighted image. Yellow contour corresponds to the area of semi-automatic segmentation of engraftment. Red contour corresponds to the area of scar as defined by 5 standard deviations from remote tissue. Blue contour corresponds to remote tissue. (D) Masson’s trichrome microscopy image of a single sample. *CE-MRI* contrast-enhanced magnetic resonance imaging.

Fig. 2 presents results for a single co-registered PDMS-matured PSC-CM treated subject, including (A, B) DTI parameter maps, (C) CE-MR for localization, and (D) a Masson’s trichrome stain of the corresponding slice using anatomical landmarks. Graft tissue is depicted as a region of signal attenuation within surrounding hyperenhancement reflecting scar in the CE-MR images. Graft areas (yellow contour) demonstrated higher levels of anisotropy than the surrounding scar tissue (red contour).

**Fig. 3:**
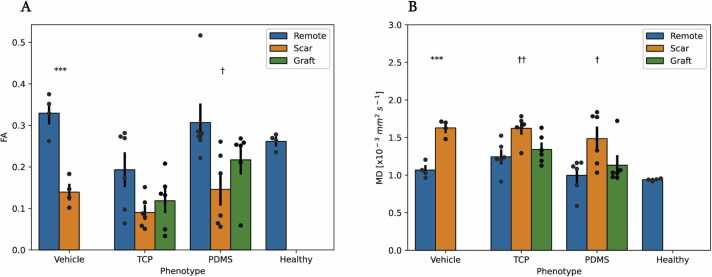
Remote, scarred, and graft territories have heterogeneous structures, with unique anisotropic signatures. (A) Fractional anisotropy (FA) values across subjects contrast healthy control, vehicle, polydimethylsiloxane (PDMS), and tissue culture plastic (TCP)-matured pluripotent stem cell-derived cardiomyocyte (PSC-CM) treated hearts. (B) Mean diffusivity (MD) demonstrated structural heterogeneity across each cell-treated group. All data here represent the mean ± standard error of the mean. ***p < 0.001 as calculated by paired t-test; ^†^p < 0.05, ^††^p < 0.01 as calculated by one-way analysis of variance (ANOVA).

Fig. 3 depicts a regional analysis based on ROIs defined from CE-MRI. As expected, the scar regions in all three cohorts demonstrated typical low anisotropy values. In particular, FA was low (0.14 ± 0.02, 0.09 ± 0.02, and 0.15 ± 0.04) while MD had correspondingly higher values (1.62 ± 0.05, 1.62 ± 0.07, and 1.48 ± 0.14 ×10^−3^ mm^2^/s) for vehicle, TCP, and PDMS-treated groups. Graft areas had an intermediate anisotropy between the remote and scarred areas (0.22 ± 0.03, 0.31 ± 0.04, and 0.15 ± 0.04), respectively, for the PDMS cohort. In addition, one-way ANOVA demonstrated significant differences between the remote, scarred, and graft regions in MD (p < 0.05) and FA (p < 0.05) of the PDMS-matured cohort. However, FA for the remote, scarred, and graft regions of the TCP treatment group only trended toward significance (p = 0.06).

**Fig. 4:**
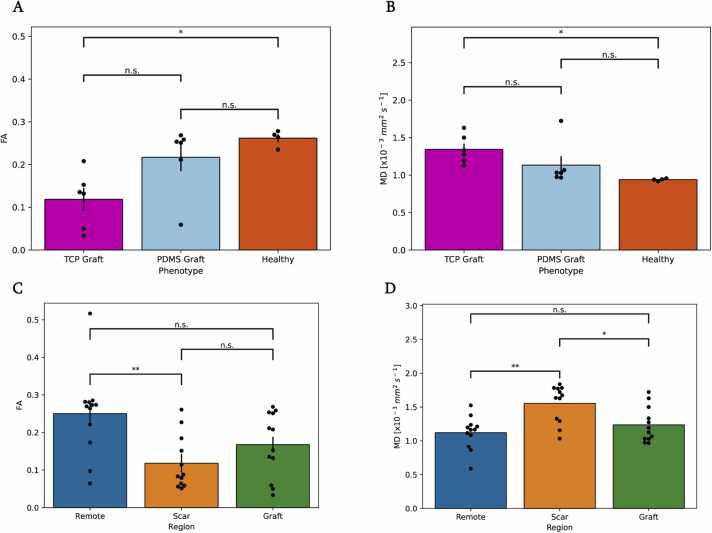
Polydimethylsiloxane (PDMS) grafts have structurally equivalent anisotropy to control myocardium. (A) Fractional anisotropy (FA) of PDMS-matured grafts showed no significant difference to healthy myocardium, while tissue culture plastic (TCP) matured grafts were significantly different than healthy myocardium. (B) Mean diffusivity (MD) of PDMS grafts was not significantly different than healthy myocardium, while TCP grafts were significantly different. (C) Scar tissue FA within the cell-treated cohort was significantly different than in remote territory. (D) Scar tissue MD within the cell-treated cohort was significantly different than in remote territory, as were scar and grafted regions. All data here represent the mean ± standard error of the mean. *p < 0.05, **p < 0.01 as calculated by one-way analysis of variance (ANOVA) with the Tukey honest significant difference post-hoc test for all timepoint pooled data.

Interestingly, FA and MD of PDMS-matured grafts were not significantly different than those for the anterior region of healthy control myocardium (p = 0.5 and p = 0.4, respectively) (Fig. 4). While not significant, PDMS-matured cells also had higher anisotropy values than their TCP-matured counterparts (0.22 ± 0.05 and 0.12 ± 0.02, p = 0.05). TCP grafts also showed significantly lower FA than the control (0.12 ± 0.02 and 0.26 ± 0.01, p < 0.05). TCP-matured grafts on the other hand exhibited significantly higher MD than the control (1.3 ± 0.08 × 10^−3^ mm^2^/s and 0.93 ± 0.01 × 10^−3^ mm^2^/s, p < 0.05), and trended higher compared to PDMS-matured grafts (1.3 ± 0.08 × 10^−3^ mm^2^/s and 1.1 ± 0.12 × 10^−3^ mm^2^/s, p = 0.2).

### 3.2 Helical and aligned architecture of PDMS-matured PSC-CM grafts

**Fig. 5:**
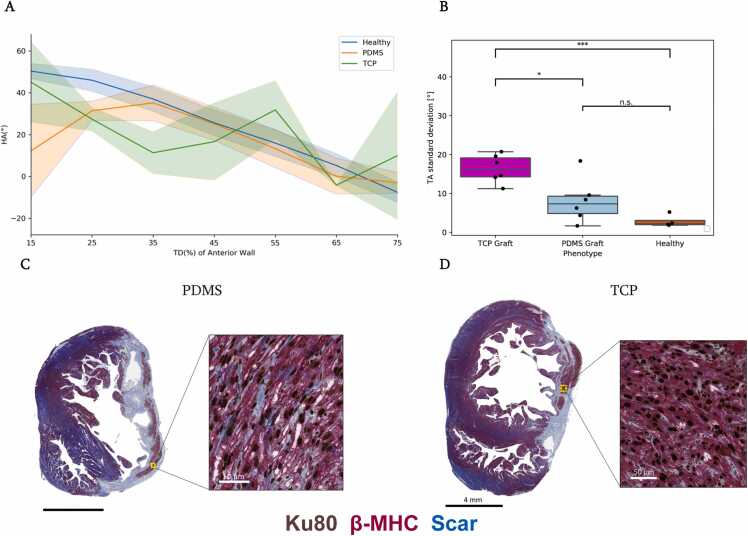
Polydimethylsiloxane (PDMS)-matured grafts are more helically and structurally aligned. (A) Binned helix angle (HA) values sampled from endo- to epicardium across the cell-treated and control cohorts. Healthy control myocyte orientations within the anterior wall, HA transmurality (HAT) = −0.93°/%TD (transmural depth) (*r* = −0.76); PDMS grafts HAT = −0.73°/%TD (*r* = −0.46); and tissue culture plastic (TCP) grafts HAT = −0.61°/%TD (*r* = −0.27). (B) Standard deviation of the transverse angle (TA) of PDMS graft was not significantly different than healthy control myocardium, while TCP-matured grafts showcased more misaligned myocytes compared to PDMS grafts and healthy controls (p < 0.05 and p < 0.001, respectively). (C) High and low magnification of Masson’s trichrome histology slide of PDMS graft. Graft was identified via immunostaining of the human nuclear marker Ku80 and host was identified via β myosin heavy chain (β-MHC). (D) High and low magnification of Masson’s trichrome histology slide of TCP graft. *p < 0.05, **p < 0.01, ***p < 0.001 as calculated by one-way analysis of variance (ANOVA) with the Tukey honest significant difference post-hoc test.

Healthy, TCP, and PDMS graft myocyte HA maps demonstrated degrees of linearity across the anterior wall. HA values were binned into 15% transmural sections. There were no significant differences between the three groups across bins (Fig. 5A). The control cohort demonstrated a steep, linear progression from RH to LH cardiomyocyte orientations, from endo- to epicardium, and high HAT (*r* = −0.76, HAT = −0.93°/%TD). PDMS grafts showcased a moderate slope and HA progression (*r* = −0.46, HAT = −0.73°/%TD) while TCP grafts demonstrated a weakly linear progression (*r* = −0.27, HAT = −0.61°/%TD).

TA of healthy controls demonstrated low variability, and hence highly aligned myocytes throughout the anterior myocardium as expected (Fig. 5B). PDMS-matured grafts exhibited aligned myocytes that were not significantly different than the healthy control (p = 0.4). The TCP-treated cohort demonstrated a degree of graft alignment that was significantly different than the PDMS (p < 0.05) and healthy cohort (p = 3 ×10^−4^). Myocytes within the PDMS-matured graft (Fig. 5C) demonstrated a more aligned structure than the disorganized TCP-matured grafts (Fig. 5D).

No differences in the number of LH, circumferential, or RH cardiomyocyte orientation voxels of vehicle vs healthy controls were found (p = 0.94, p = 0.55, and p = 0.55, respectively). Likewise, differences in HAT within the remote territory were not found between cell-treated groups (p = 0.15).

**Fig. 6:**
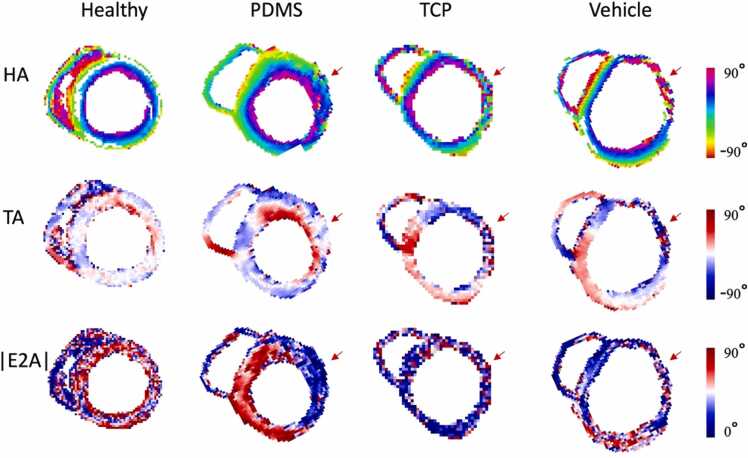
Scar of vehicle-treated subjects is disorganized, while cell-treated subjects have more aligned and helical grafts. Top row: helix angle (HA) maps of one representative short-axis slice of the healthy control, polydimethylsiloxane (PDMS)-matured cell, tissue culture plastic-matured cell, and vehicle-treated cohorts. group. Middle row: transverse angle (TA) maps of each cohort. Bottom row: absolute value of secondary eigenvector angle (E2A) maps of each cohort. Graft/scar regions are demarcated with the red arrow.

Representative slices of HA, TA, and E2A maps across the four cohorts are shown in Fig. 6. Treatment and scarred regions highlighted with a red arrow show disorganization within the primary direction of the myocytes, as expected. Qualitatively, the degree of disorganization within the treated cohort, specifically the PDMS-matured group, is less pronounced.

**Fig. 7:**
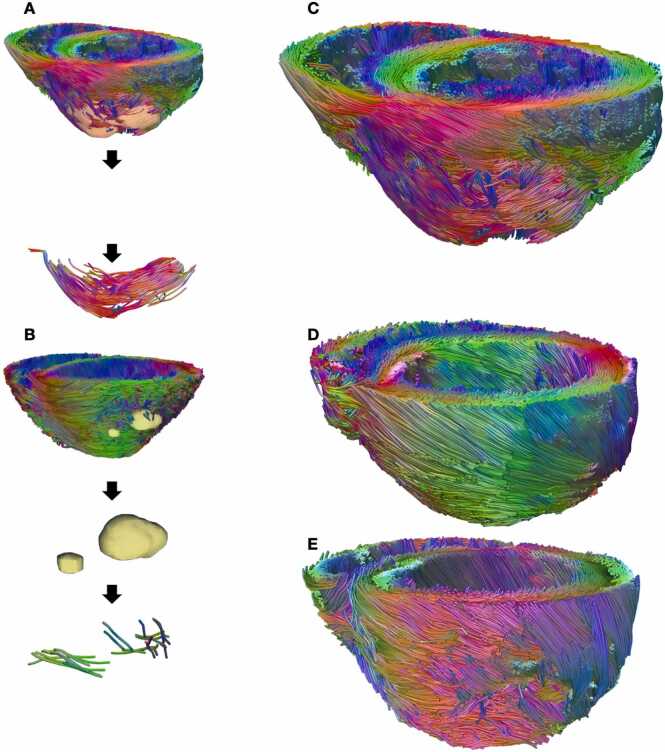
Graft 3D rendering showcases aligned PSC-CM structure. (A) Workflow of 3D engraftment segmentation begins with semi-automated segmentation of engraftment using CE-MR image. Segmentation is then imported into DSI Studio, where a segmentation mask is used to initialize tractography. Blue tracts correspond to longitudinal myocyte orientations, while red and green tracts correspond to an arbitrary x-y coordinate system. Polydimethylsiloxane (PDMS) graft exhibits helical orientation with elongated and aligned myocyte orientations. (B) Tissue culture plastic-matured graft exhibits disorganized myocyte orientations across adhered tissue. (C) Whole heart tractography of PDMS-matured pluripotent stem cell-derived cardiomyocyte-treated subject in graphic A. (D) Whole heart tractography of healthy control showcasing left-handed myocyte orientations on epicardium, transitioning to right-handed myocyte orientations on endocardium. (E) Vehicle heart tractography demonstrating lack of myocyte cohesion in the area of anterolateral ablation territory. *3D* three-dimensional, *PSC-CM* pluripotent stem cell-derived cardiomyocytes, *CE-MR* contrast-enhanced magnetic resonance.

Tractography of a PDMS-matured graft (Fig. 7) qualitatively corroborates the findings of Fig. 5. PDMS graft myocyte orientations are aligned and exhibit a degree of helicity about the long axis (Fig. 7A). Tractography of TCP-matured graft exhibits more disorganized structure in two unconnected graft regions (Fig. 7B). Fig. 7D demonstrates the expected LH to RH helical myocyte orientation transmurally throughout the myocardium. Fig. 7E showcases a vehicle-treated subject with a pronounced loss of epicardial tissue in the area of cryoablation.

### 3.3 Histology vs DTI myocyte graft alignment

Measurements of graft myocyte angles between DTI and histology showed good correlation (*r* = 0.82, *R*^2^ = 0.57, Additional File 1). Bland-Altman analysis depicted good agreement between the two measurements, with a −2.40° negative bias.

## 4 Discussion

To the best of our knowledge, this is the first study to demonstrate direct detection and structural quantification of engrafted PSC-CMs using non-invasive MRI. Several studies have used DTI and CE-MRI to investigate structural improvements in the host myocardium post-therapy; however, direct detection of the therapeutic agent within the host was not observed. In line with these studies, we used several MR imaging biomarkers to test the hypothesis that PSC-CM matured grafts will exhibit a more natural structural phenotype via tissue anisotropy and primary cardiomyocyte alignment.

PDMS-matured PSC-CMs have increased elongation, a more organized sarcomeric structure, and a higher cell area than their TCP counterparts [8]. DTI biomarkers consistently demonstrated PDMS grafts have a more adult-like phenotype. For instance, markers of anisotropy, including FA and MD, demonstrated PDMS grafts were not significantly different than healthy tissue and trended toward higher anisotropy than TCP grafts. Structural parameters of PDMS grafts, such as HAT and TA variability, showcased an overall improved cell alignment compared to TCP grafts.

Vehicle-treated cohorts showcased the expected decrease in anisotropy and myocyte coherence within the scarred territory. Interestingly, no changes in left-handed, circumferential, or right-handed myocyte orientations were found in the remote territory of the vehicle cohort. Similar studies using DTI to investigate remodeling post-MI have found loss of RH primary cardiomyocyte orientation voxels within the endocardial remote territories at the 8- and 13-week post-MI timepoint in porcine models [13], [25]. This was not seen in our study. Another study in mice found FA to increase in remote territories post-MI [26], which was found in our study (Fig. 3). Imaging biomarkers of left ventricular (LV) remodeling in the companion study [8], including increased LV end-systolic and end-diastolic diameters as measured by echocardiography, were also pronounced in the vehicle cohort. Moreover, they found attenuation of LV end-systolic and end-diastolic diameters in the cell-treated cohorts compared to vehicle-treated subjects. The cell treatment may, therefore, have a positive effect in suppressing the degree of LV remodeling. Interestingly, no changes in LH, circumferential, or RH cardiomyocyte orientations were found in our study. One possible reason may be due to the cryoablation model of injury. Other studies employed an ischemic insult via coronary balloon catheterization and found a loss of endocardial RH cardiomyocyte orientation. As opposed to endocardial damage, cryoablation is done on the epicardium which will induce different structural stressors throughout the LV. Therefore, the mechanics of remodeling for the cryoablation model of MI would not necessarily mimic those of the ischemic injury model. It is important to note that the cryoinjury model employed in this study has a different mechanism of damage than ischemia, which primarily affects the endocardium, unlike cryoablation which targeted the epicardium in this study. Further studies investigating PSC-CM therapy post-MI would therefore be important in determining the degree of structural remodeling.

Gadolinium has been shown to affect diffusion parameters. For instance, increased FA and decreased MD have been observed in the areas of gadolinium accumulation in brain tissue particularly in the area of contrast enhancement [27]. Diffusion of the gadolinium contrast agent over time may explain the elevated MD in our study compared to others. Here, care was taken to scan the subjects shortly after fixation was complete to ensure minimal gadolinium was diffused from the scar. Moreover, this effect would have been seen in all samples as they were all scanned within a similar imaging timeline. It has also been shown that prolonged formalin fixation on cardiac tissue alters diffusion properties, specifically increasing MD in tissues stored for 6–8 weeks [28]. In addition, diffusion parameters can vary based on chosen diffusion sequence parameters, fixation time, and gadolinium use which may explain why our MD values are intermediate to those previously reported [28], [29]. Therefore, the experimental parameters should be considered when comparing studies.

Variation in remote FA was found in two samples of the TCP group. We speculate this may be due to variable degrees of hypertrophy in these samples, as they were specifically from the 2-week post-transplantation cohort. We also found lower FA within the graft region of Fig. 4A in the TCP cohort, presumably because TCP grafts are immature and have non-statistically lower anisotropy than their PDMS-matured counterparts. TCP scar does seem to have lower FA than vehicle scar FA, but we speculate this is due to sample variance. Although each 3D volume was inspected for image quality, variation within groups does exist and may also account for this bias.

Cardiac DTI tractography has been used in numerous applications, with associated quantitative measurements being used to detect ischemic myocardium [30] and qualitatively to visualize the myocardium post-cell therapy [12]. Here, we present the first study to showcase the application of CMR tractography to visualize exogenous PSC-CMs in a host myocardium. The PDMS graft showcased qualitatively good correspondence to structural characteristics of healthy myocardium, that is, circumferentially aligned myocyte orientations based on TA variability. However, it remains to be seen how, or if, the grafted cells reorient themselves in the in vivo setting based on the surrounding dynamic environment of the beating myocardium. Hence, it would be interesting to quantify the longitudinal time course of cell reorientation using DTI tractography of engrafted PCM-CMs in future work.

As the cardiac contractile state was not accounted for during the fixation process, cardiomyocyte directionality parameters are more heterogeneous.

## 5 Limitations

A limitation of this study was obtaining adequate contrast between scar and healthy cardiomyocytes post-gadolinium injection. Due to the dispersion of the contrast agent at the fixation stage, some subjects had no detectable scar. This limited our ability to identify grafts based on our inclusion criteria, and hence data were omitted from the study. Although in vivo demonstration of this technique has proven feasible [6], more robust methods of cell tracking are warranted for the consistent localization of grafts using CMR. A second limitation is that the cryoablation model does not represent clinical MI. A small sample size was a limitation of this study with animals sacrificed at different time points. The destructive nature of tissue microscopy herein limited our ability to validate 3D findings. Other image modalities, to confirm such findings, should be implemented in future studies, such as computed tomography. Although post-treatment scar sizes did not differ among experimental groups, post-injury scar sizes before cellular or sham cocktail treatment were not accounted for and hence indirect treatment benefits must be considered.

## 6 Conclusions

Here, we have shown that CMR is a useful non-invasive tool to interrogate imaging biomarkers of PSC-CM graft structure. Future studies employing cardiac DTI may help parse the relationship between structural characteristics of PSC-CM grafts and remodeled myocardium, and how they affect functional recovery within the heart.

## Funding

This research was funded by the University of Toronto’s Medicine by Design/Canada First Research Excellence Fund initiative, the McEwen Stem Cell Institute, the Peter Munk Cardiac Center, the Canada Research Chairs Program, and the Ted Rogers Centre for Heart Research.

## Author contributions

Conceptualization of the study: M.C., N.R.G., G.A.W., and M.A.L.; developing the CMR methodology: N.R.G., G.A.W., and M.C.; development and execution of animal and cell therapy: W.D. and M.A.L.; formal CMR analysis: M.C.; financial resources: N.R.G., G.A.W., and M.A.L.; data curation: M.C. and W.D.; writing—original draft preparation: M.C.; writing—review and editing: M.C., N.R.G., G.A.W., W.D., and M.A.L.; preparation of figures: M.C.; supervision: N.R.G., G.A.W., and M.A.L.; project administration; N.R.G., G.A.W., and M.A.L.; funding acquisition: N.R.G., G.A.W., and M.A.L. All authors have read and agreed to the published version of the manuscript.

## Ethics approval and consent

All procedures were approved under the Animal Care Committee of the University Health Network.

## Consent to publication

Not applicable.

## Declaration of competing interests

The authors declare the following financial interests/personal relationships which may be considered as potential competing interests: Michael Laflamme reports a relationship with BlueRock Therapeutics that includes consulting or advisory and equity or stocks. Wahiba Dhahri has patent #US App. No. 16/701,734 pending to N/A. Michael Laflamme has patent #US App. No. 16/701,734 pending to N/A. The other authors declare that they have no known competing financial interests or personal relationships that could have appeared to influence the work reported in this paper.

## Appendix A Supplementary material

Additional File 1: Histology validates DTI cardiomyocyte orientation measurements across graft structure. (A) Each point represents DTI cardiomyocyte orientation angle mean and histological graft ROI measurements, where a line simulating perfect agreement is included for reference to showcase degree of correlation. (B) Bland-Altman plot depicting the relationship among measurements. Coefficient of determination (*R*^2^) and Pearson's correlation coefficient (*r*) showcase good agreement and correlation.
