# Supplementary figures and images for: Using diffusion tensor imaging to depict myocardial changes after matured pluripotent stem cell-derived cardiomyocyte transplantation
Source: J Cardiovasc Magn Reson. 2024 May 23;26(2):101045. doi: 10.1016/j.jocmr.2024.101045 (PMC11278291; doi:10.1016/j.jocmr.2024.101045)

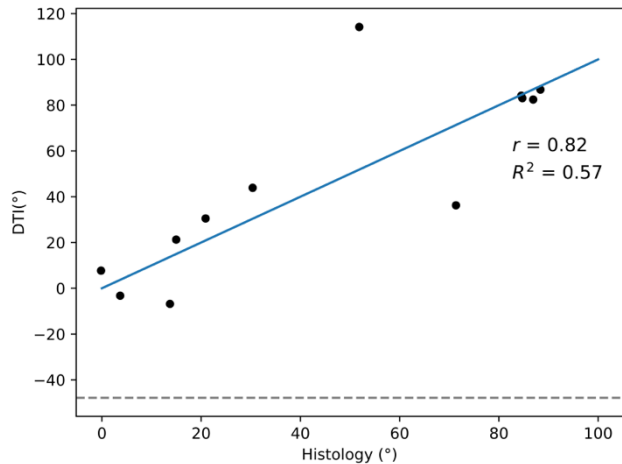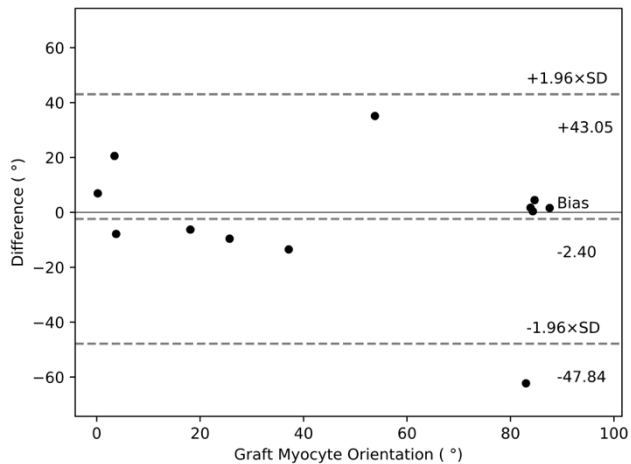

Supplement: Supplementary file 1 — Additional File 1: Histology validates DTI cardiomyocyte orientation measurements across graft structure. (A) Each point represents DTI cardiomyocyte orientation angle mean and histological graft ROI measurements, where a line simulating perfect agreement is included for reference to showcase degree of correlation. (B) Bland-Altman plot depicting the relationship among measurements. Coefficient of determination (R2) and Pearson's correlation coefficient (r) showcase good agreement and correlation. [file mmc1.pdf]
